# Magnitude of CD8 cell reactivity with a peptide pool is comparable to the sum of responses by individual peptides

**DOI:** 10.1186/2051-1426-1-S1-P94

**Published:** 2013-11-07

**Authors:** Tameem Ansari, Wenji Zhang, Ioana Moldovan, OLeg Targoni, Ramu A Subbramanian, Paul V Lehmann

**Affiliations:** 1 R&D, Cellular Technology Ltd., Shaker Hts., OH, USA

## Introduction

Immune monitoring of T cell responses increasingly relies on the use of peptide pools. Peptides within the pool that are restricted by the same HLA allele, however, will compete for HLA binding displacing each other. Therefore, how much T cell reactivity goes undetected when one works with peptide pools?

## Methods

Using a model peptide pool that is comprised of 32 well-defined viral epitopes from Cytomegalovirus, Epstein-Barr virus, and Influenza virus (CEF peptide pool), we assessed peptide competition in PBMC from 43 human subjects. IFN-γ was measured using the ImmunoSpot® Test Kit, and to assure low background, serum-free, CTL-Test™ Medium was used. The spots were counted using an ImmunoSpot® S6 Core reader.

## Results

The magnitude of the peptide pool-elicited CD8 T cell responses was a mean 79% and a median 77% of the sum of the CD8 T cell responses elicited by the individual peptides. Therefore, while the effect of peptide competition was evident, it was of a relatively minor magnitude. By studying the dose-response curves for individual CEF peptides, we show that several of these peptides are present in the CEF-pool at concentrations that are orders of magnitudes in excess of what is needed for the activation threshold of the CD8 T cells.

## Conclusions

The inhibitory effects due to peptide competition were found to be relatively low for the CEF peptide pool. The presence of T cells with high affinity for the viral peptides is the reason for this relatively minor competition within the CEF pool.

